# Gastrointestinal stromal tumour of the stomach mimicking a hepatic tumour: a case report

**DOI:** 10.1186/1756-0500-7-147

**Published:** 2014-03-14

**Authors:** Duminda Subasinghe, Bowlanegedara Nimal Rathnasena, Udayanga S Medagodahetti, Thevaraj Bhishman

**Affiliations:** 1 General Surgical Unit, The National Hospital of Sri Lanka, Colombo, Sri Lanka; 2 51,3rd Lane, Negombo Road, Kurunegala, Sri Lanka

**Keywords:** Gastrointestinal stromal tumour, Gastric, Hepatocellular carcinoma, Laparoscopy, Endoscopy, Gastric

## Abstract

**Background:**

Gastrointestinal stromal tumours are the commonest mesenchymal tumours of the gastrointestinal tract. The stomach and small intestine are the favoured sites of occurrence. The symptoms of these depend on the site, size of the tumour and may include abdominal pain, gastrointestinal bleeding or signs of obstruction. We describe a woman with extra luminal gastrointestinal stromal tumour of the stomach that mimicked a left hepatic tumour presenting as an abdominal mass.

**Case presentation:**

A 51-year-old woman presented with a history of increasing epigastric pain for two-months duration. Her contrast-enhanced computed tomography of the abdomen revealed a large mass in relation to left lobe of the liver. On laparoscopy there was a large tumour arising from the lesser curvature of the stomach. The tumour was resected and histology was suggestive of gastrointestinal stromal tumour.

**Conclusion:**

This case shows new evidence for the presentation of extra luminal gastric gastrointestinal stromal tumour that are very rare and can mimic hepatic tumour.

## Background

Gastrointestinal stromal tumours (GISTs) are rare [1], but represent the most common type of mesenchymal neoplasms that arises from the gastrointestinal (GI) tract. They account for 1–3% of all gastric neoplasms, 20% of all small bowel tumours, and 0.2– 1.0% of all colorectal tumours [2-4]. Symptoms due to GIST are not typical and depend on the localization and the tumour size. About 10-30% of GIST are completely asymptomatic, and are discovered accidentally during the endoscopy or radiological evaluation as well as during surgical interventions performed for various other reasons. GIST usually appears in patients above 50 years of age, whereas the maximum incidence is observed in the 5th and the 6th decade of life. The mean age at the diagnosis is 55–63 years [5]. The diagnostic evaluation of GIST is based on imaging techniques, with a special role of endoscopic examination, because it is commonly accessible, however the most important diagnostic tools are the histological and immunohistochemical examinations.

Some gastric sub mucosal tumours that are considered to be gastrointestinal stromal tumor (GIST). Extra gastric compression may mimic the symptoms and endoscopic findings of gastric submucosal tumours. Computed tomography (CT) scan can accurately differentiate extra gastric compression from true sub mucosal tumours. However, cases may arise that cannot be differentiated even after various methods are used. We describe a Sri Lankan female patient with an extra luminal GIST presented as an abdominal mass which was misdiagnosed as a hepatocellular carcinoma. She underwent various investigations including endoscopy and abdominal CT scan.

## Case presentation

**Figure 1:**
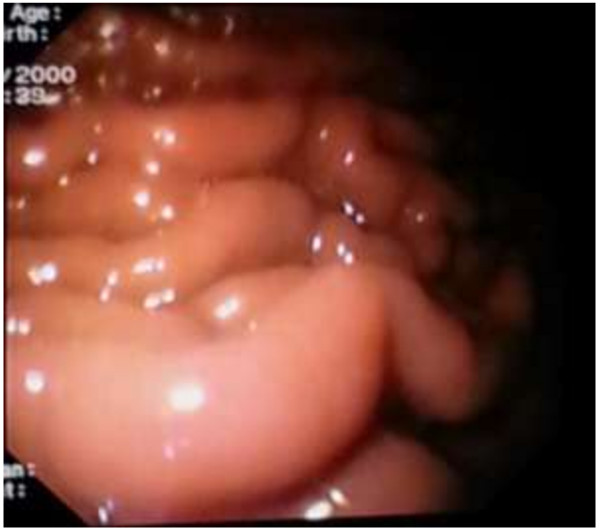
Upper gastrointestinal endoscopy showing normal mucosa of stomach.

**Figure 2:**
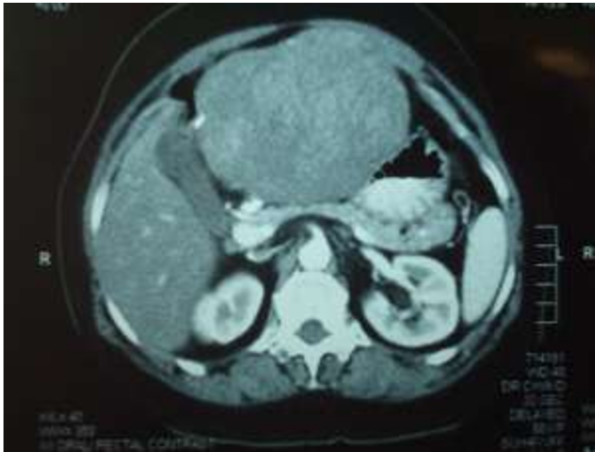
CT Scan abdomen showing the mass.

**Figure 3:**
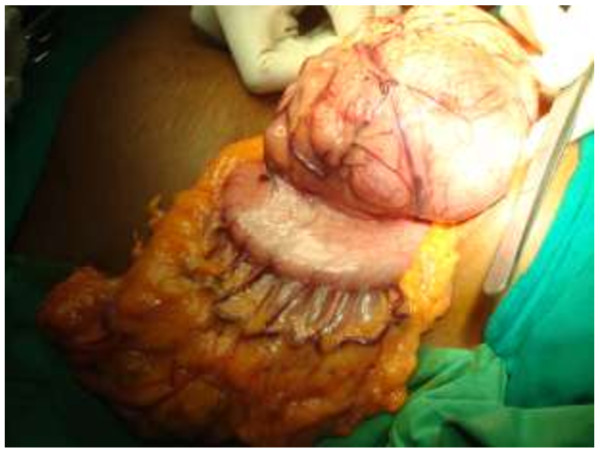
Tumour arising from lesser curvature of stomach.

**Figure 4:**
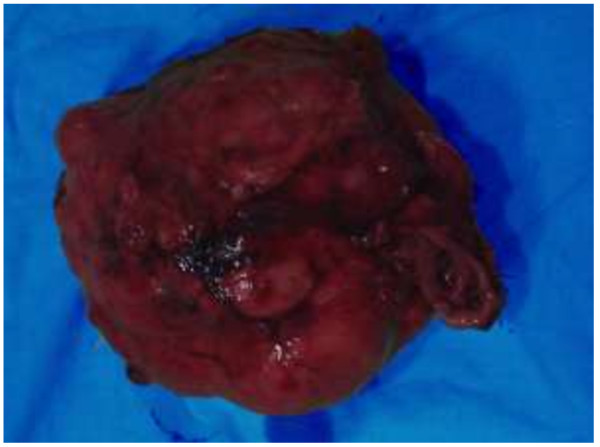
Resected surgical specimen of GIST.

**Figure 5:**
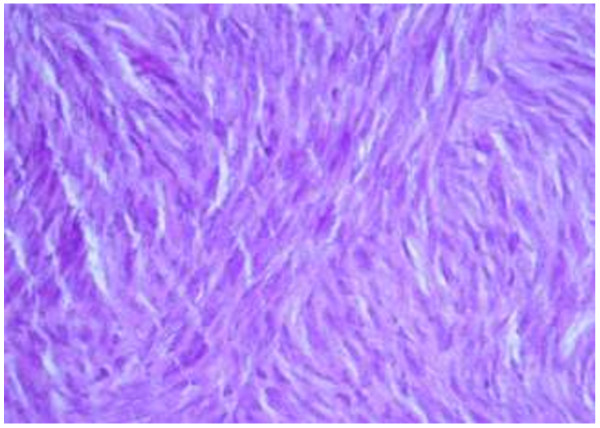
Histology showing spindle cells of GIST.

A 51-year-old Sri Lankan woman presented with epigastric pain for two months. Initial examination showed that she had tenderness in the epigastrium. There was associated loss of appetite as well. Her past medical, surgical and familial histories were unremarkable. Routine laboratory data on admission did not show any abnormal findings. Upper gastrointestinal endoscopy was normal up to 2nd part of the duodenum and revealed a normal mucosa of the stomach (Figure 1). Abdominal CT scan showed a mass lesion (12×8×11 cm) of the left lobe of the liver with enhancement during arterial phase and washout during venous phase. The impression of the radiologist was a hepatocellular carcinoma, most probably of fibrolamellar type due to star sign within the lesion (Figure 2). The operative plan was laparoscopy and left hemi hepatectomy. Laparoscopic exploration was performed for under general anaesthesia. On the laparoscopic examination, the appearance of the liver was completely normal. There was a large solid mass arising from the lesser curvature of the stomach. There were no regional lymph node enlargement. There were no peritoneal deposits and also pelvic organs were appeared normal on laparoscopy. Then patient underwent open surgery which involved ligation of the left gastric artery and separation of the tumour from the lesser curvature (Figure 3) with a small cuff of the stomach. The weight of the surgical specimen was 0.92 kg. Later histology of the surgical specimen (Figure 4) was suggestive of a GIST). The patient had an uneventful postoperative course and was discharged after seven days on orals. The histopathological examination of the surgical specimen (Figure 5) revealed a tumour which composed of interlacing fasicles of spindle cells with wavy nuclei which are fairly monomorphic. There was no evidence of tumour necrosis. The mitotic activity of the tumour was 2 per 50 high power fields. It showed diffuse CD117 positivity in membrane and cytoplasm. The overlying gastric mucosa was histologically unremarkable and resection margins were free of tumour. The overall features were suggestive of GIST. She was not given postoperative chemotherapy. The patient was asymptomatic for past 2 years and currently been followed up as an outpatient in the clinic.

### Discussion

Gastrointestinal stromal tumours are believed to be originated from the interstitial cells of Cajal, a pace-maker cell that controls gastrointestinal peristalsis. The clinical manifestations of GISTs depend on the location and size of the tumours. Patients may present with pain, dysphagia, weight loss, gastrointestinal bleeding, bowel obstruction, or a palpable abdominal mass [6]. Surgical resection is the most effective treatment option for GIST. The 5 year survival rate after surgery amounts to 28–65% [6-8]. Tumour resection is the treatment of choice for GISTs. According to Lupescu *et al.* CT abdomen is sufficient to diagnose the location, extension, size, contours, structure of the tumours, hepatic metastasis of GIST [9]. Thus, it is important to be able to diagnose GISTs from pre-operative CT. According to Rosch *et al.* the sensitivity and specificity of endoscopy are 87% and 29%, respectively for distinguishing intramural lesion from extramural compression [10]. According to the best of authors knowledge there were no published case reports describes GIST of stomach mimicked hepatic tumour. But there are reports on extra gastro intestinal GIST involving lesser omentum [11-14]. Baskiran *et al.*[15] described a large pedunculated GIST of posterior gastric wall mimicked a pancreatic tumour. Our patient’s upper gastro intestinal endoscopy showed a normal gastric mucosa. Therefore we didn’t plan for a upper GI endosonography for this patient. Here, our preoperative diagnosis was not GIST of stomach. Radiologically the wall of the stomach was not thickened and there was no intramural tumour in the gastric wall. Park *et al.* has described a left hepatic cyst may rarely mimic a submucosal tumour arising from the gastric cardia and fundus [16]. Our patient had a GIST of a stomach mimicking left hepatic tumour. In the best of our knowledge, this is the first report of a patient with gastric extra mucosal GIST that was misdiagnosed as a hepatocellular carcinoma of left lobe of liver and diagnosed by laparoscopy and treated by surgery.

## Conclusion

We conclude that extra mucosal gastric GISTS are rare and can present as mass abdomen. An Extra mucosal GIST of stomach may mimic a tumour arising from the left lobe of liver and cause nonspecific abdominal symptoms. For such a case, laparoscopic procedure is a useful option for making the accurate diagnosis and treatment.

## Consent

Written informed consent was obtained from the patient for publication of this case report and accompanying images. A copy of the written consent is available for review by the Editor-in-Chief of this journal.

## Competing interests

The authors declare that they have no competing interests.

## Authors’ contributions

DS and BGNR were involved in the preparation of this manuscript. BGNR, USM, BT, DS performed the operation. All were involved in the post operative care of the patient. All authors read and approved the final manuscript.

## Authors’ information

BGNR is a Senior Consultant General Surgeon, DS is a Registrar in Surgery, USM is a Registrar in Surgery, BT is a Senior Registrar in General Surgery.
